# Short-Term Soil Flushing with Tannic Acid and Its Effect on Metal Mobilization and Selected Properties of Calcareous Soil

**DOI:** 10.3390/ijerph18115698

**Published:** 2021-05-26

**Authors:** Zygmunt Mariusz Gusiatin, Joeri Kaal, Agnieszka Wasilewska, Jurate Kumpiene, Maja Radziemska

**Affiliations:** 1 Department of Environmental Biotechnology, Faculty of Geoengineering, University of Warmia and Mazury in Olsztyn, 10719 Olsztyn, Poland; agniwasi@gmail.com; 2 Pyrolyscience, 28015 Madrid, Spain; joeri@pyrolyscience.com; 3 Department of Civil, Environmental and Natural Resources Engineering, Lulea University of Technology, 97187 Lulea, Sweden; jurate.kumpiene@ltu.se; 4 Faculty of Civil and Environmental Engineering, Institute of Environmental Engineering, Warsaw University of Life Sciences, 02776 Warsaw, Poland; maja_radziemska@sggw.edu.pl

**Keywords:** heavy metals, soil column, tannic acid sorption

## Abstract

Cadmium, Cu, Ni, Pb, and Zn removal via soil flushing with tannic acid (TA) as a plant biosurfactant was studied. The soil was treated for 30 h in a column reactor at a constant TA concentration and pH (3%, pH 4) and at variable TA flow rates (0.5 mL/min or 1 mL/min). In the soil leachates, pH, electrical conductivity (EC), total dissolved organic carbon, and metal concentrations were monitored. Before and after flushing, soil pH, EC, organic matter content, and cation exchange capacity (CEC) were determined. To analyze the organic matter composition, pyrolysis as well as thermally assisted hydrolysis and methylation coupled with gas chromatography-mass spectrometry were used. Metal fractionation in unflushed and flushed soil was analyzed using a modified sequential extraction method. The data on cumulative metal removal were analyzed using OriginPro 8.0 software (OriginLab Corporation, Northampton, MA, USA) and were fitted to 4-parameter logistic sigmoidal model. It was found that flushing time had a stronger influence on metal removal than flow rate. The overall efficiency of metal removal (expressed as the ratio between flushed metal concentration and total metal concentration in soil) at the higher flow rate decreased in this order: Cd (86%) > Ni (44%) > Cu (29%) ≈ Zn (26%) > Pb (15%). Metals were removed from the exchangeable fraction and redistributed into the reducible fraction. After flushing, the soil had a lower pH, EC, and CEC; a higher organic matter content; the composition of the organic matter had changed (incorporation of TA structures). Our results prove that soil flushing with TA is a promising approach to decrease metal concentration in soil and to facilitate carbon sequestration in soil.

## 1. Introduction

Two of the most important anthropogenic contaminants that are commonly found in soils are heavy metals/metalloids and hydrophobic organic pollutants such as polycyclic aromatic hydrocarbons (PAHs). In contrast to PAHs, metals and metalloids persist in the soil for a long time, e.g., 150–5000 years for Pb [1]. Globally, metals and metalloids have contaminated soil in over 5 million sites covering 20 million ha of land [2]. This widespread pollution is due to the fact that metals and metalloids are in soils in different concentrations and from various sources including mining, smelting, military training, electronics industries, fuels, waste disposal, and agriculture [3]. 

Among different remediation methods, ex situ soil washing and in situ soil flushing have been shown to have great potential for removing a wide range of contaminants from soils including various metals and metalloids [4]. Although metals are usually removed more efficiently from acidic soils than from calcareous soil [5,6], both types of soil can be remediated with soil washing or soil flushing [5]. However, there are more reports in the literature on soil washing than on soil flushing. This may be related to the fact that soil washing usually removes pollutants more efficiently than soil flushing. Both methods differ in hydrodynamic conditions [7]. In soil washing, the soil is thoroughly mixed with the washing solution, whereas in soil flushing, the solution is percolated through the soil. However, soil flushing has the advantages that it consumes less washing agent and has lower operating costs than soil washing [8]. In contrast to soil washing, soil flushing does not require excavating, handling, and transporting large quantities of contaminated soil and has the potential to be used in combined remediation systems [9]. Soil flushing is performed as a continuous cycle, which reduces consumption of water and flushing agents. After it is used for treatment, the flushing solution is reinjected into the contaminated soil, circulated, reextracted, and repurified [10]. The cost of soil flushing was estimated to be in the range of 20–104 USD/m^3^ soil, whereas that of soil washing was 70–200 USD/m^3^ soil [2,11].

Soil washing and soil flushing solutions can consist of water, acidic aqueous solutions, basic solutions, or complexing agents, reducing agents, or surfactants [2]. Up to now, ethylenediaminetetraacetic acid (EDTA) and its salts have been most extensively tested for the remediation of metal contaminated soils [12,13,14,15,16]. Soil flushing has been practiced more commonly to remove organic pollutants by flushing surfactant solutions through contaminated soil [2]. Soil flushing with synthetic surfactants (Triton, Tween) and citric acid has also been tested for simultaneous removal of metals and PAHs from spiked sandy soil (pH 7.3, 0.12% organic matter) in China [17,18]. Additionally, microbial rhamnolipids have been used in soil flushing of acidic Pb–Zn mine tailings (as sandy soil in texture, pH 3.6, 4.7% organic matter) collected from Bathurst, New Brunswick (Canada) [19]. It has been shown that soil flushing with rhamnolipid foam can be even more effective than flushing with a rhamnolipid solution because foam spreads and penetrates the soil (even heterogenous soils) more effectively than a solution. 

Our previous studies have demonstrated that plant biosurfactants have great potential for removal of metals with saponin from three types of soils (i.e., loamy sand, loam, silty clay, pH 6.1–7.2, 1.6–10.3% organic matter) spiked with Cu, Cd, and Zn [20] and for removal of arsenic (As) with tannic acid (TA) from soils (silty loam in texture, pH 4.4–7.9, 5.9–18.1% organic matter) in areas formerly used for As mining and smelting in Zloty Stok (Poland) [21]. Although plant biosurfactants are used less commonly than rhamnolipids, and TA is used less commonly than saponin, TA offers a number of advantages, namely, its low cost and nontoxicity [22]. TA is a naturally occurring plant polyphenol that is a type of hydrolyzable tannin, composed of a glucose moiety with numerous esterified gallic acid units. It has a large complexing capacity, even among hydrolysable tannins, due to the abundance of adjacent hydroxyl groups on the gallic acid moiety [23]. It has a wide range of applications in areas such as food, medicine, dye processing, water purification technology, and corrosion inhibition. TA is capable of reducing Fe (III) to Fe (II) ions. When Fe (II) is complexed to TA, it is unable to participate in Fenton-like processes and to mediate the formation of hydroxyl radicals [24]. There is little information on the use of TA in soil remediation and, moreover, only concerning soil washing [21,25,26] and phytoremediation [27], not soil flushing. 

Our previous studies have indicated that TA has potential as a washing agent. It has removed Cu, Pb, and Zn from soil (as silty loam, pH 6.8, 0.21% organic matter) in an industrial area around the Legnica Copper Smelter in the Lower Silesia (Poland) with an efficiency similar to that of saponin [25]. It has also removed As (arsenic) from soils collected in Zloty Stok (Poland) with an efficiency of 50–64% [21]. However, taking into account the cost of plant biosurfactants and the fact that less washing solution is consumed in soil flushing than in soil washing, it may be even more desirable to use TA as a flushing agent. Despite this potential economic advantage of soil flushing with TA, its use as a flushing agent (column experiments) has not been investigated.

The aim of this study was to determine the effect of TA on the simultaneous mobilization of Cd, Cu, Ni, Pb, and Zn from calcareous soil by column flushing at two flow rates of TA solution (i.e., 0.5 and 1.0 mL/min). Based on previous results from batch tests with TA [25], short-term soil flushing was tested in this preliminary experiment. In column leachates, pH and electrical conductivity (EC) were measured to identify how they can affect the release of metal from the soil as well as total organic carbon (TOC) to monitor TA concentration during soil flushing. The effect of soil flushing with TA on selected soil properties and metal distribution was also determined. Additionally, the organic matter in the soil, before and after flushing, was characterized by analytical thermal techniques, i.e., pyrolysis coupled with gas chromatography-mass spectrometry (Py–GC–MS) and thermally assisted hydrolysis and methylation coupled with gas chromatography-mass spectrometry (THM–GC–MS). This was mainly done to identify the changes induced by flushing with TA and to determine whether the TA was incorporated among soil constituents.

## 2. Materials and Methods

### 2.1. Model Soil

The soil was collected from an agricultural area in Warmia and Mazury Province, northeastern Poland. The soil was taken from an area of 5 m^2^. A total sample (approximately 3 kg) consisted of 5 site sub-samples that were mixed to obtain an average soil sample. After transportation to the laboratory, the soil was air-dried, milled, sieved through a 2 mm sieve, and characterized. The unspiked soil was sandy loam with an alkaline pH (8.5) and low organic matter content (4.1%). The total metal concentrations (mg/kg) in this soil were as follows: 0.0 (Cd), 6.6 (Cu), 4.8 (Ni), 0.9 (Pb), and 16.8 (Zn). Next, the soil was spiked with a mixture of five metals (i.e., Cd, Cu, Ni, Pb, and Zn) by adding an aqueous solution (1 L) containing 0.0549 g Cd(NO_3_)_2_·4H_2_O, 1.4641 g Cu(NO_3_)_2_·2.5H_2_O, 0.9863 g Ni(NO_3_)_2_·6H_2_O, 1.5985 g Pb(NO_3_)_2_, and 3.6385 g Zn(NO_3_)_2_·6H_2_O to 2 kg of soil. Then, the soil was incubated at room temperature and a constant moisture (60% of maximum water holding capacity) for 1 month. After spiking, the soil was air-dried, crushed, and kept in tightly closed plastic containers for further experiments. The physico-chemical characteristics of the spiked soil are given in Table 1. 

In our preliminary study, we simulated the elevated metal concentrations that can be found in agricultural soil affected by industrial emissions (Cd, Pb) and application of fertilizers and pesticides (Cd, Cu, Ni, Zn) [28]. The most toxic of these metals (Cd, Pb) can be easily absorbed by crops and be incorporated in the food chain. All metal concentrations exceeded the permissible values for agricultural soils according to Polish law [29]. 

### 2.2. Flushing Agent 

**Figure 1:**
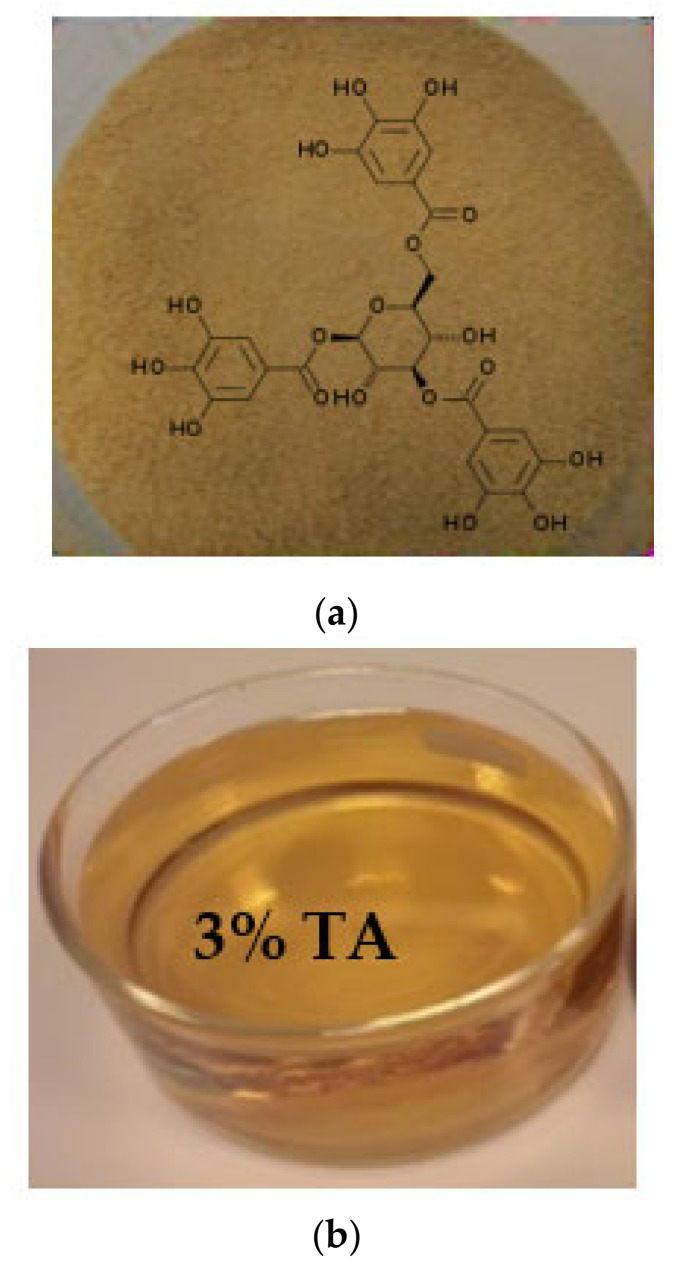
Tannic acid (TA) used as a soil flushing agent: (**a**) TA powder; (**b**) TA solution.

As a flushing agent, 3% TA was used (Figure 1, product No. 16201, Sigma–Aldrich, Burlington, MA, USA). TA is a natural polyphenolic compound (C_76_H_52_O_4_) that is in the form of a powder with a molecular weight of 1701.2 g/mol. The concentration of TA for soil flushing was selected based on previous batch soil washing experiments [21]. The original 3% (m/v) TA solution had a pH of 4.0 ± 0.2, a surface tension of 41.5 mN/m, and a density of 1.085 g/mL.

### 2.3. Soil Flushing Process

**Figure 2:**
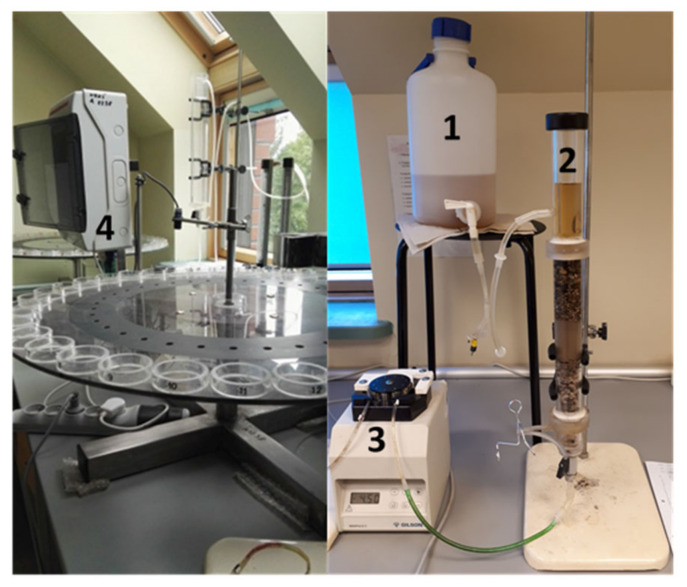
The setup for soil flushing: container for the flushing solution (1); column reactor (2); peristaltic pump (3); automatic sample collector (4).

The system for soil flushing (under dynamic conditions) consisted of a column reactor, a container for flushing solution, a peristaltic pump, an automatic sample collector, and an automatic device for collecting samples (Figure 2). 

A cylindrical plexi column (total length 30 cm, diameter 3 cm) was filled with contaminated soil. From top to bottom, the column reactor was packed with the following layers, with the thickness of each layer given in brackets: thicker gravel φ 2–4 mm (4 cm), fine gravel φ 1–2 mm (4 cm), soil layer (8.5 cm), fine gravel φ 1–2 mm (4 cm), and thicker gravel φ 2–4 mm (4 cm). At the bottom of the column, nylon mesh was placed to prevent the loss of soil particles from the column. The flushing solution was supplied from the top to the bottom of the column, with the peristaltic pump at one of two flow rates (0.5 mL/min or 1.0 mL/min) and at a hydraulic gradient of 1.3–1.5. Before the flushing solution was supplied to the soil column, the column was pre-wetted with distilled water to fill the empty pores. The leachate from the soil column was continuously supplied to the automatic sample collector. All of the leachate that was collected for 1 hour was considered to be one sample. In total, soil flushing was conducted for 30 h, and 30 samples were collected. The experiment was repeated twice. 

In the leachate from the soil column, the concentrations of Cd, Cu, Ni, Pb, and Zn were measured, along with the pH, EC, and TOC. Before measurement of metal concentrations and TOC, the leachates were filtered through a 0.45 µm filter. After flushing, the soil was air-dried and milled to determine the fractionation of the metals, selected physico-chemical characteristics of the soil (i.e., pH, EC, organic matter content, cation exchange capacity) and organic matter composition using the Py-GC-MS and THM-GC-MS techniques.

### 2.4. Analytical Methods

Soil particle size distribution was determined using a Mastersizer 2000 particle size analyzer (Malvern Panalytical Ltd, Malvern, UK). The water holding capacity of the soil was determined by the amount of water held in the soil sample versus the dry weight of the sample. The soil bulk density was determined as a ratio between the dry weight of the soil and the volume of the soil. The equilibrium soil pH and EC was measured in distilled water (1:2.5 ratio, m/V) with a pH meter (Hanna HI 221, Hanna Instruments, Woonsocket, RI, USA) and a conductometer (Hanna HI 8733, Hanna Instruments, Woonsocket, RI, USA), respectively. Soil organic carbon was analyzed using the Tiurin method. This is a wet combustion of soil organic carbon with 0.4 M potassium dichromate solution at boiling point for 5 min in the presence of Hg_2_SO_4_. The excess of dichromate was titrated with Mohr’s salt solution. The soil organic matter was recalculated as organic carbon × 1.724 [30]. The cation exchange capacity (CEC) of the soil was determined as the sum of the hydrolytic acidity (in 1N Ca (CH_3_COO)_2_) and exchangeable bases (in 0.1 M HCl) according to Kappen’s method [30]. The concentration of TOC in TA solution and in column leachates was measured using a Shimadzu Liquid TOC-VCSN analyzer (Shimadzu Corporation, Kyoto, Japan).

Total contents of Cd, Cu, Ni, Pb, and Zn in soil, before and after soil flushing, were measured with a flame atomic absorption spectrometer (FAAS) (AA 280FS, Varian, NSW, Australia). The soil after drying was digested in a mixture of 12.2 mol/L HCl and 14.5 mol/L HNO_3_ at a volume ratio of 3:1 in a microwave oven (MARSXpress, CEM, Matthews, NC, USA) for 45 min at 170 °C. TraceCERT® heavy metal standards for FAAS (Sigma–Aldrich, Saint Louis, MO, USA) were used to prepare the calibration curve. The accuracy of metal analysis by FAAS was validated by analyzing the reference material, CRM 142 R. The concentrations of metals that were recovered were satisfactory, ranging from 95% to 104%. The limits of detection (LOD) for individual metals were 0.07, 0.39, 0.03, 1.60, and 0.29 mg/L for Cd, Cu, Ni, Pb, and Zn, respectively. The limits of quantification (LOQ) were 0.21, 1.19, 0.10, 4.84, and 0.87 mg/L for Cd, Cu, Ni, Pb, and Zn, respectively.

The distribution of the metals in the soil was determined using a modified BCR (Commission of the European Communities Bureau of Reference) procedure [31] in which metals were fractionated into four fractions using specific reagents: exchangeable and acid-soluble (mobile F1) extracted with 0.11 mol/L CH_3_COOH; reducible (potentially mobile F2) extracted with 0.5 mol/L NH_2_OH·HCl; oxidizable (potentially mobile F3) extracted with 1 mol/L CH_3_COONH_4_; residual (immobile F4) extracted with HCl and HNO_3_ mixed at a volume ratio of 3:1. Based on metal fractionation, the mobility factor (MF) was calculated [7] to compare metal mobility in soil before and after soil flushing. All physico-chemical analyses were performed in triplicate.

For analytical pyrolysis, soil samples before and after flushing were treated with 2% HF solution (50 mL aqueous HF: 1.0 g soil, overnight shaking). The HF treatment was performed three times (each time decanting the supernatant after centrifugation at 2000 rpm for 5 min), followed by three times of washing with distilled water. The residue was dried at 35 °C to obtain the final residue. For conventional analytical pyrolysis (i.e., pyrolysis-gas chromatography-mass spectrometry (Py-GC-MS)), approximately 1 mg of sample was embedded with quartz wool into quartz tubes. The tube was inserted into the Pt-filament probe of a CDS 5000 Pyroprobe (CDS Analytical, Oxford, MS, USA), and pyrolyzed for 20 s at 650 °C (heating rate 10 °C/ms). The pyrolysate was analyzed by a coupled Agilent 6890/5975 GC-MS system (Agilent Technologies, Santa Clara, CA, USA). For details on the temperature program of the GC and other parameters, please refer to Kaal et al. [32]. In addition, the samples were analyzed by thermally assisted hydrolysis and methylation (THM-GC-MS), using the same method but with the addition of 25% aqueous tetramethyl ammonium hydroxide (TMAH, Sigma–Aldrich, Saint Louis, MO USA) prior to pyrolysis. This kind of analytical pyrolysis is also referred to as thermochemolysis. Pure TA (product No. 16201, Sigma–Aldrich, Munich, Germany) as received was analyzed as well. Information on the THM-GC-MS of tannins, including TA reference material, can be found in Nierop et al. [33]. The chromatograms were evaluated qualitatively, and no analytical replicates were conducted.

The data on cumulative metal removal were analyzed using OriginPro 8.0 software (OriginLab Corporation, Northampton, MA, USA). The data were described with a 4-parameter logistic sigmoidal model: R_cm_ = d + (a-d)/(1 + (t/c)b), where R_cm_ is the cumulative metal removal at a specific flow rate of TA (mg/kg), a is the theoretical response at time zero, b is the slope factor, c is the mid-range time (inflection point), and d is the theoretical response at infinite time. 

The statistical significance of differences between the values of selected soil characteristics before and after soil flushing with TA were determined with Tukey’s HSD test (Statistica 13.3, StatSoft).

## 3. Results

### 3.1. Characteristic of Leachates from Soil Flushing with TA

**Figure 3:**
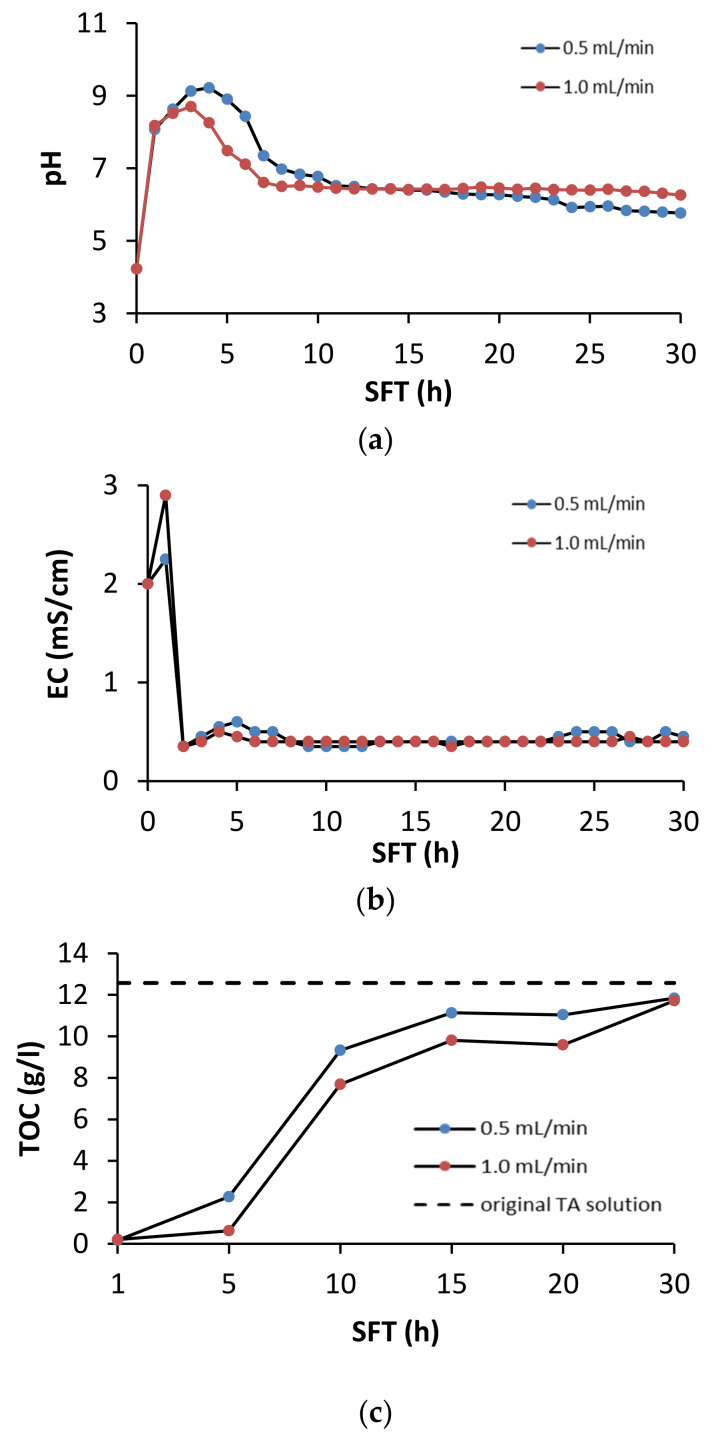
Effect of soil flushing time (SFT) on (**a**) pH, (**b**) EC and (**c**) dissolved TOC in TA leachate from the column reactor.

During soil flushing, the pH, EC, and TOC concentrations were monitored in the leachates from the column reactor. The changes in these characteristics are shown in Figure 3.

The greatest changes in the pH of the leachate took place during the first 7 h of soil flushing (Figure 3a). After the first hour of soil flushing at both flow rates, the pH in the leachate increased considerably, and then it reached its maximum after 4 h of flushing at a flow rate of 0.5 mL/min and after 3 h at 1.0 mL/min This suggests that changes in the chemistry of the leachate were dynamic. Because the original pH in the TA solution was acidic (pH 4.0), it favored the release of alkaline cations from the calcareous soil, resulting in a sudden increase in the pH of the leachate. Due to the fact of its acidic nature, TA can efficiently extract Ca and Mg from soil [34]. Between the 10th and 30th hour of soil flushing, the pH in the leachate stabilized (Figure 3a). With the use of EDTA as a flushing agent, Di Palma et al. [30] found that, due to the soil buffer properties, the pH of leachate from soil flushing was approximately 7.5 when the flushing solution was used at pH 5 and approximately 8.5 when the initial pH of the flushing solution was 8.0. The TA solution had a relatively high electrolytic conductivity. At both flushing rates, its conductivity quickly increased to a value higher than that in the original solution, but then it decreased to values lower than the starting value (Figure 3b). 

In both variants, the TOC concentration in the TA solution dropped quickly, reaching a minimum after 1 hour of the process (Figure 3c). Then, it steadily increased to close to the original value by the end of the process. These results suggest that TA can be easily and quickly retained on soil particles under soil flushing conditions. TA sorption occurred to the greatest extent during the first 10 h of soil flushing. After the soil was saturated with TA, its concentration in the leachate stabilized and was close to the concentration in the original solution. The tendency of TA to sorb to soil can be a critical determinant of metal mobilization, as this tendency limits its ability to form soluble extractable complexes with metals [34]. A decrease in flushing solution concentration is typical during soil flushing. Di Palma et al. [35] reported that the loss of EDTA concentration (as TOC) during flushing of calcareous soil was 83% at a flow rate of 1.2 mL/min and 81% at a flow rate of 10.2 mL/min. 

### 3.2. Metal Removal during Soil Flushing with TA

**Figure 4:**
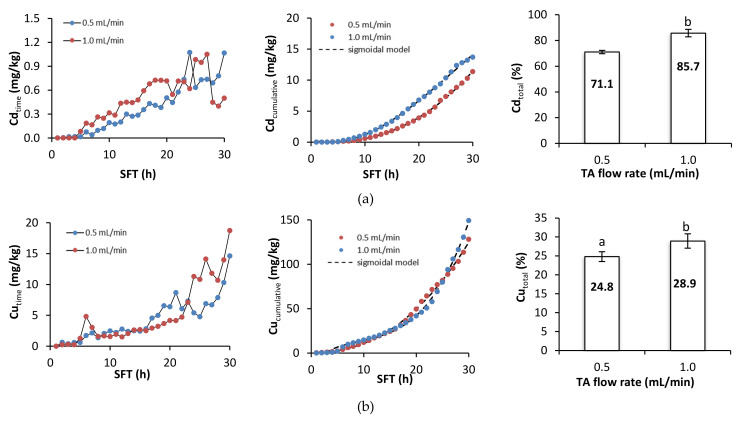

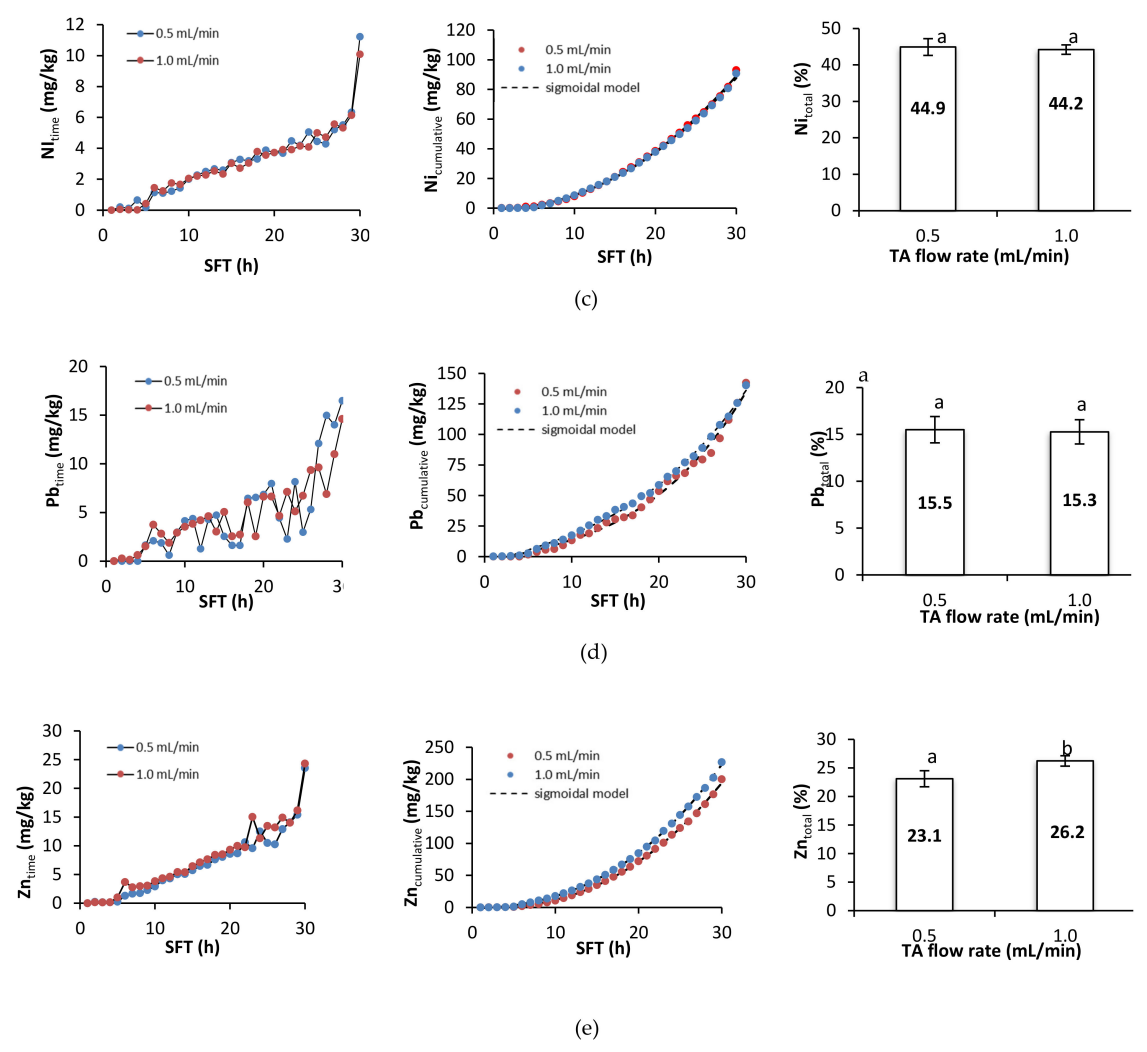
Metal removal, cumulative metal removal (in mg/kg), and metal removal efficiency (in %) from soil as a function of soil flushing time (SFT) and TA flow rate: (**a**) Cd, (**b**) Cu, (**c**) Ni, (**d**) Pb, and (**e**) Zn. For metal removal efficiency, different letters indicate significant differences in metal removal between the two flow rates (ANOVA followed by Tukey’s honest significant difference test, *p* < 0.05).

Figure 4 presents metal removal at specific times during soil flushing, cumulative metal removal, and the total efficiency of metal removal. During the first hours of soil flushing, little or no metal was removed at both flushing rates. Depending on the specific metal, cumulative metal removal began to increase visibly somewhere between the 5th and the 10th hour of soil flushing, and it continued to increase until the end of the experiment. The total efficiencies of removal of Cd, Cu, and Zn were significantly higher at a flow rate of 1.0 mL/min than at one of 0.5 mL/min, but the total efficiencies of removal of Ni and Pb did not differ significantly between the flow rates. Although the amounts of each metal removed at a specific flushing time tended to increase as the experiment progressed, there were some fluctuations and temporary deviations from this trend. Fluctuations in Pb removal were high throughout the process; Cd and Cu removal also displayed some fluctuations, and there were sudden increases in Ni and Zn removal near or at the end of soil flushing, which suggests that 30 h of soil flushing with TA may be insufficient for effective removal of these two metals.

A sigmoidal model was fitted to the experimental data on cumulative metal removal. The *R*^2^ coefficients for the model ranged between 0.9901 and 0.9996, indicating a good fit. The low values of the standard error (SE, Table 2) suggest that the estimates of the parameters were precise.

To summarize, metal removal during the first 5–6 h of soil flushing was negligible. With the starting pH of the soil at approximately 8.2, free carbonates were dissolved prior to metal removal. This was also highly likely to be related to the high sorption of TA during the first hours of soil flushing (a decrease in TA concentration by 95% at 0.5 mL/min and by 82% at 1.0 mL/min). High sorption of TA was also confirmed by the Py-GC-MS and THM-GC-MS analyses (Section 3.4). A higher flow rate had a positive effect on soil flushing, particularly with regard to the removal of Cd, Cu, and Zn. These results are in agreement with results obtained from soil flushing with other chelators. For example, Di Palma et al. [35], during treatment of alkaline soil (pH 8.8) in a column reactor with 0.05 M EDTA, obtained the highest Cu removal efficiency (93%) at pH 5 and a flow rate of 1.2 mL/min. Similarly, Maity et al. [36], when treating soil (pH 7.8) in a column reactor with foam from a saponin solution at a concentration of 0.15 g/l, showed that the efficiency of Cu (95%), Pb (98%), and Zn (56%) removal was highest at a pH of four and a flow rate of 1 mL/min. On the other hand, despite the large shares of metals in the exchangeable fraction, leaching of these metals at the beginning of the process could be limited by Ca, Mg, and Na, which may be leached more dynamically than heavy metals at the start of flushing [37].

The cumulative efficiency of removal of all metals increased visibly when the flushing time was extended. Based on the changes in cumulative removal efficiency over time, it can be concluded that 30 h was not sufficient to obtain equilibrium conditions. Assuming that metal removal would continue at the same rate if the flushing time were extended, it can be estimated via linear extrapolation that equilibrium removal would be reached within 71–136 h (at 0.5 mL/min) or 62–133 h (at 1.0 mL/min). Thus, soil flushing with TA could last for 3–6 days. Soil flushing with chelators can require a relatively long time. For example, Hauser et al. [38] found that metals (Cu, Zn, Pb) flushing from soil with EDDS took as much as a few weeks. 

This suggests that soil treatment with TA under flushing conditions may depend more on the flushing time than on the flow rate of TA. This tendency was observed by Liu et al. [39], who removed Cu and Pb from soil under dynamic conditions, feeding an 8% potassium lignosulfonate solution to the reactor at a flow rate of 1–1.5 mL/min. As the flushing time was increased (0–6 h), Cu removal efficiency increased from approximately 2% to 23%, and the efficiency of Pb removal increased from approximately 0.5% to 20%. Other authors indicate that efficient metal removal under dynamic conditions may require a relatively long soil flushing process. Maity et al. [36] flushed soil for 24–72 h and observed that metal removal efficiency increased as the process was lengthened, although equilibrium conditions were not achieved. Arwidsson et al. [40] demonstrated that metal removal by amino polycarboxylic acids would require leaching to last as long as several days to be efficient. In the case of TA, the conditions of soil remediation (soil washing or soil flushing) can be important with regard to the duration of soil treatment, and they can be more important for soil washing than for soil flushing. Gusiatin [41] found that when using TA under batch conditions, an extraction time of 24 h is appropriate for As removal from brownfield soils. 

### 3.3. The Effect of Soil Flushing with TA on Metal Redistribution

The efficiency of soil flushing depends upon the capability of the flushing agents to solubilize or chelate metals, the properties of the soil, and the distribution of the metals in the soil [17]. In contaminated soil, Cu, Cd, Zn, and Ni prevailed in the exchangeable fraction (Table 3) in which metals are usually electrostatically bound or co-precipitated with carbonates [25]. The alkaline pH of the contaminated soil favored metal co-precipitation with carbonates. The exception was Pb, which was present mainly in the reducible fraction. The high content of most metals in the F1 fraction indicated that the metals were bioavailable and mobile, posing a threat to the environment [42]. The least available metals are in the residual fraction [36], but their share in the studied soil was small. In a study by Kuziemska et al. [43] with soil spiked with Ni (100 mg/kg) and fractionated by the BCR method, 74% of Ni was bound in the exchangeable fraction, and only 7.4% of Ni was in the residual fraction. The data presented here show that a high proportion of metals in the most mobile fraction is typical of soils contaminated by anthropogenic sources.

Short-term soil flushing with TA changed the metal distributions, principally with regard to the F1 and F2 fractions. metals were removed mainly from the exchangeable fraction (F1). The efficiency of metal removal from this fraction varied from 39.6% to 87.8% at a flow rate 0.5 mL/min and from 43.4% to 86.1% at a flow rate of 1.0 mL/min. A higher TA flow rate facilitated metal removal from the F1 fraction, with the exception of Pb, which was removed from the F1 fraction with similar efficiency at both flow rates (Table 3). Due to the structure of tannins, they can complex metals with hydroxyl groups and pi-cation binding sites [44]. TA is a weak organic acid, and its dissociation strongly depends on the pH. At pH 4, the dissociation degree of TA is 7.1·10^−2^, which corresponds to approximately 10% ionization of TA [45]. Thus, proton replacement from hydroxyl groups seems to be the main mechanism of metal removal under soil flushing conditions [46]. 

In flushed soil, the concentration of most metals (except for Ni and Cd at 1.0 mL/min) in the reducible (F2) fraction was higher than that in unflushed soil (Table 3). This indicates that Fe and Mn oxides played a crucial role in metal redistribution. This is interesting because TA displays reducing properties due to the fact of its naturally acidic pH and the numerous phenolic groups in its structure. These phenols take part in redox reactions by forming quinones and donating electrons [47]. Phenolic compounds are known to rapidly mobilize or solubilize soil metals, such as Ca, Al, and Fe, probably through chelation and oxidation/reduction reactions [34,48,49]. The reducing capacity of TA is greater in acidic than in alkaline conditions [50]. Redox potential regulates the distribution and bioavailability of metals in soils. Metals in the F2 fraction are associated with amorphous Fe/Mn (oxyhydr)oxides, which are readily released in reductive conditions.

Thus, TA can reduce Fe- and Mn-oxides and release metals that are coprecipitated with or sorbed onto these oxides, decreasing the metal content in the reducible fraction. However, in the present study, the content of metals in the reducible fraction increased, which was probably due to the fact of two causes. Firstly, under soil flushing conditions, the reduced Fe (II) might have been oxidized to Fe (III) (oxyhydr) oxides, which have a strong affinity for heavy metals [51], and metals extracted from the exchangeable fraction could have been partially readsorbed onto oxides. Secondly, it has been reported that TA is sorbed onto inorganic soil constituents such as metal oxides [52,53]. Thus, sorption of TA with previously complexed metals onto metal oxides could have increased the pool of metals in the reducible fraction in the flushed soil. 

Under flushing conditions, TA did not remove metals from the oxidizable fraction at either flow rate, with the exception of Cu. In the flushed soil, the metal concentration in the F3 fraction was higher than that in the unflushed soil (Table 3). This indicates that TA caused the loss of soil organic matter, but it was sorbed onto native organic matter in the soil. The incorporation of TA–metal complexes into soil organic matter was reflected by an increase in metal concentrations in the oxidizable fraction. These results indicate that the use of some organic washing agents can increase the share of metals in the oxidizable fraction in the soil. Kulikowska et al. found that application of dissolved organic matter (DOM) from sewage sludge for Cu, Pb, and Zn removal from soil via batch washing increased the metal content in the more stable fractions (oxidizable and residual), which may have resulted from DOM sorption in soil and, as a result of this, redistributed some soluble metals into more stable fractions [54].

In contrast to its effect on the F3 fraction, TA showed some ability to remove metals from the residual (F4) fraction (Table 3). It should be noted that the concentrations of metals in the F4 fraction were relatively low (from 14.9 to 58.2 mg/kg). Although the strength of metal bonding in the F4 fraction in spiked soil is lower than that in real contaminated soil, it has been shown that, during soil washing, TA partially removes Cu and Zn from the F4 fraction in soil affected by the smelting industry [25]. 

### 3.4. The Effect of Soil Flushing with TA on Soil Properties

The principle aim of soil flushing of metal-contaminated soil is to reduce the total concentration, bioavailability, and mobility of metals. However, characterization of soil properties is also important for further soil management. After soil flushing with TA, changes in some soil properties were observed. The changes in selected soil characteristics are shown in Table 4. 

In general, the flow rate of TA had more of an influence on metal content and mobility than on other soil properties such as pH, EC, organic matter, and CEC. Soil treatment with TA decreased the soil pH from alkaline to slightly acidic (from 8.19 to 6.7 on average), indicating an increase in soil acidity. Unlike other polymeric polyphenols, TA is quite acidic because it contains substantial amounts of gallic acid, and it can increase the extractability from soil of alkaline elements such as Ca and Mg [34]. Halvorson et al. [55] demonstrated that addition of gallic acid decreased soil pH by approximately 1–2 pH units, presumably protonating cation exchange sites and mobilizing metals like Ca. 

In the study, the electrical conductivity in soil decreased from 2.1 to 0.7–0.8 mS/cm after flushing, meaning that TA decreased soil salinity from slightly saline to non-saline. 

Despite an increase in soil organic matter, the CEC in flushed soil was lower than that in unflushed soil. This decrease is attributable to differences between the pH of soil (8.2) and that of TA (4.0). The acidic pH of TA could result in protonation of carboxyl and hydroxyl sites on soil constituents (e.g., organic matter, metal oxides) and, thus, reduce the CEC [56]. 

TA can sorb to soil thus affecting the content and quality of soil organic matter. In this study, the organic matter content was two-fold higher after flushing with TA at either flow rate than in the unflushed soil. Sorption of polyphenol compounds in soil is usually due to the fact of hydrogen bonding and hydrophobic interactions [49] or bi- and tridentate inner sphere complexes. The latter mechanism is stimulated by the presence of reactive mineral phases, such as poorly crystalline Fe (oxy) (hydr)oxides, as their effect on surface area and microporosity facilitates tannin entrapment and covalent binding [57]. Tannins can take part in the formation of humic-like substances, as reactive quinones from tannins can self-polymerize or co-polymerize with other compounds such as amino-containing compounds [53]. Thus, sorption of TA can be an opportunity for carbon sequestration in remediated soil. 

**Figure 5:**
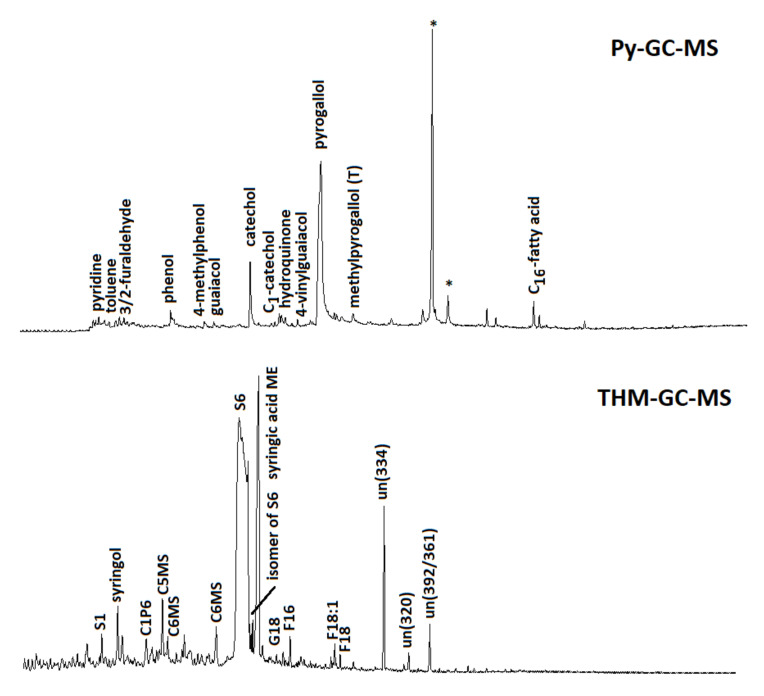
Pyrolysis-GC-MS (above) and THM-GC-MS (below) total ion current chromatograms of soil samples after flushing with TA. The large peaks for catechol and pyrogallol (Py-GC-MS) and 3,4,5-trimethoxybenzoic acid methyl ester (S6; THM-GC-MS) indicate the presence of TA after flushing. Asterisks mark contamination (from sample handling and the analytical system). Other abbreviations for THM-GC-MS: S1 = 1,2,3-trimethoxybenzene; C1P6 = 4-methoxy-x-methyl-benzoic acid methyl ester); C5MS and C6MS indicate methylated forms of metasaccharinic acids (from carbohydrates); G18 = methylated ferulic/caffeic acid methyl ester; F16 and F18 are C16 and C_18_ fatty acid methyl esters; C18:1 = monounsaturated C_18_ fatty acid (oleic acid methyl ester). The compounds marked “un” are identified (numbers between parentheses indicate characteristic *m*/*z* fragments).

The characterization of soil organic matter with the Py-GC-MS chromatogram (Figure 5) revealed that the unflushed soil sample provided a fingerprint that was typical of largely plant-derived soil organic matter, i.e., a series of alkanes/alkenes from aliphatic biopolymers and waxes, methoxyphenols (mostly guaiacols), and 4-vinylphenol (from p-coumaric acid) from lignin, probably mainly from herbaceous plants [58]. 

In addition, polysaccharide products (furans and furaldehydes), N-containing pyrolysis products from proteins (indoles, diketopiperazines), and other phenols (phenol and methylphenols) can originate from plant and microbial sources (e.g., [59]). After soil flushing, the pyrolysate was radically different, evidenced by a dominance of catechols and pyrogallols, clearly indicating the retention of TA by the soil and perhaps its incorporation into the soil organic matter. Indeed, these compounds are known as the main products of TA and of other tannins with a trihydroxybenzene configuration [60], including the TA used here and analyzed separately. The soil samples after flushing did not produce a peak that could be allocated to the carbohydrate nucleus of TA but neither did the untreated pure TA sample, which may indicate a high degree of galloylation or chromatographic bias. Potential differences in TA’s molecular features with and without interaction with the soil cannot be addressed reliably due to the completely different matrix composition of the samples (80% minerals in soil after flushing, 0% in pure TA), and even despite the HF treatment, this has major effects on reaction dynamics and due to the large (flattened) and broad peaks of, especially, the pyrogallol moieties. By THM-GC-MS, similar observations can be made, but the TA derivatives are now recognized as di- and trimethoxybenzenes, in particular 3,4,5-trimethoxybenzoic acid methyl ester (methylation product of gallic acid; [33]). The lignin present in the organic matter of the soil sample (before or after flushing) was characterized by the methylation products of p-coumaric and ferulic (or caffeic) acids (P18 and G18), showing that it was indeed largely grass-derived and in a good preservation state. Fatty acid methyl esters (FAMEs), including methoxy-substituted FAMEs, could be detected as well. Their chain length distribution suggests that they originated from plant materials.

Hence, the pyrolysis analyses showed unambiguously that large amounts of TA are present in the soil after flushing, which was probably related to the major shift in the color of the sample due to the flushing as well (from yellowish to brownish). The data suggest that the TA actually overshadowed the organic matter that was native to the soil sample, but it must be acknowledged that the data have a qualitative character (the multiple esteried gallic acid units were efficiently released during THM-GC-MS, possibly causing a positive bias).

Short-duration soil flushing with TA decreased the total Cd concentration (at a flow rate of 1.0 mL/min) and the total Ni concentration (at both flow rates) in soil below the national permissible limits for soil in agricultural areas (Table 1 and Table 4). Decreasing heavy metal concentrations is a crucial objective for remediation of soil for its future reuse. Much more attention should be paid to the environmental risks posed by the presence of mobile metal fractions [61]. The advantage of using TA is that it decreases metal mobility (MF), defined as the ratio of the metal concentration in the F1 fraction to the sum of the metal concentrations in the F1, F2, F3, and F4 fractions [20]. Based on the MF, metal in soil can be considered immobile (<1%), weakly mobile (1–10%), moderately mobile (11–30%), highly mobile (31–50%), or very highly mobile (>50%) [7]. On the basis of this classification, metals in the unflushed soil were highly and very highly mobile. With this classification, TA decreased the mobility of Cu and Pb to moderately mobile and weakly mobile, respectively. Considering that, after flushing, Ni, Cd, and Zn remained highly mobile, and that the cumulative removal of these metals tended to increase as the flushing time increased, it can be assumed that extended flushing times may be required to further decrease the mobility of these metals.

## 4. Conclusions

The present study assessed the efficiency of short-term flushing with TA of soil co-contaminated with Cd, Cu, Ni, Pb, and Zn. Metal removal was influenced to a greater extent by the type of metal and the duration of soil flushing than the flow rate of TA. Cumulative metal removal visibly increased between the 5th and 30th hours of flushing, but equilibrium conditions were not obtained. This means that the effectiveness of TA may be greater if the flushing time is extended. Regardless of the soil flushing conditions, the shares of all metals in the exchangeable fraction decreased, and their shares in the reducible fraction increased, probably due to the changes in redox potential conditions and sorption of TA on soil constituents. Soil flushing with TA decreased soil pH, salinity, and cation exchange capacity but increased the content of organic matter two-fold. The incorporation of TA into soil organic matter was confirmed by Py-GC-MS and THM-GC-MS analyses. 

Short-term soil flushing with TA is important for decreasing the environmental risk posed by metals in soil and for increasing the share of stable organic matter in soil, which can serve as a precursor for the formation of humic substances. TA displays the potential for use in soil remediation and restoration. Further assessments during long-term soil flushing of metal removal with TA and its effects on soil properties will be necessary.

## Figures and Tables

**Table 1:** Selected physico-chemical characteristics of the spiked soil (mean ± SD, *n* = 3).

Characteristic	Unit	Value	Acceptable Values ^a^
Sand	%	48.7	-
Silt	%	44.6	-
Clay	%	6.7	-
Bulk density	g/mL	1.16	-
Water holding capacity	%	62.2	-
pH	-	8.2 ± 0.4	-
Electrical conductivity	mS/cm	2.1 ± 0.2	-
Organic matter	%	4.1 ± 0.7	-
Cation exchange capacity	cmol/kg	47.8 ± 2.7	-
Total Cd	mg/kg	16.0 ± 1.3	3
Total Cu	mg/kg	515.8 ± 8.9	150
Total Ni	mg/kg	245.1 ± 3.8	150
Total Pb	mg/kg	919.1 ± 14.6	250
Total Zn	mg/kg	865.8 ± 10.1	500

^a^ Acceptable values according to regulations by the Polish Ministry of the Environment [29] for agriculture areas.

**Table 2:** The values of the standard errors (SEs) of the parameters of the sigmoidal models.

Flow Rate/Metal	Cd	Cu	Ni	Pb	Zn
0.5 mL/min	0.110	1.976	1.005	2.131	1.701
1.0 mL/min	0.147	1.383	0.926	1.682	1.488

**Table 3:** Concentrations of metals in individual fractions in unflushed and flushed soil (SFT = 30 h) and the efficiency of metal removal (in %) from individual fractions (mean ± SD, *n* = 3).

Metal	Fraction *	Unflushed Soil	Flushed Soil			
			0.5 mL/min	1.0 mL/min	0.5 mL/min	1.0 mL/min
		mg/kg	mg/kg	mg/kg	%	%
**Cu**	F1	264.3 ± 8.3 a	94.7±5.9 b	67.2 ± 3.5 c	64.2	74.6
	F2	164.3 ± 3.1 a	176.0±6.1 b	203.4 ± 9.6 c	−7.1 **	−23.8
	F3	30.9 ± 0.9 a	31.5±1.3 a	35.5 ± 1.6 b	−1.9	−14.9
	F4	56.3 ± 2.6 a	53.5±3.9 a	52.8 ± 4.1 a	9.0	6.2
**Ni**	F1	167.3 ± 6.2 a	93.0±3.1b	80.6 ± 4.7c	44.4	51.8
	F2	53.2 ± 1.8 a	40.3±3.6 b	44.8 ± 2.3 b	24.2	15.8
	F3	9.7 ± 1.1 a	11.4±1.8 b	11.1 ± 2.2 b	−17.5	−14.4
	F4	14.9 ± 0.9 a	11.1±1.6 b	10.6 ± 1.1 b	25.5	28.8
**Cd**	F1	13.8 ± 0.7 a	4.0±0.3 b	2.6 ± 0.3 c	71.0	81.1
	F2	1.9 ± 0.2 a	2.0 ± 0.2 a	0.9 ± 0.1 b	−5.3	52.6
	F3	0.3 ± 0.1 a	0.4 ± 0.1 a	0.4 ± 0.1 a	−33.3	−33.3
	F4	0.0 ± 0.0 a	0.0 ± 0.0 a	0.0 ± 0.0 a	0.0	0.0
**Pb**	F1	313.1 ± 11.3 a	38.3 ± 3.7 b	43.4 ± 2.4 b	87.8	86.1
	F2	558.6 ± 16.1 a	636.4 ± 18.1 b	695.1 ± 15.2 c	−13.9	−24.4
	F3	31.5 ± 2.1 a	49.6 ± 3.0 b	46.4 ± 3.9 b	−57.5	−47.3
	F4	15.8 ± 1.9 a	0.0 ± 0.0 b	0.0 ± 0.0 b	100	100
**Zn**	F1	586.8 ± 17.1 a	336.2 ± 10.5 b	331.8 ± 11.6 b	39.6	43.4
	F2	206.0 ± 9.8 a	231.2 ± 11.3 b	265.7 ± 10.9 c	−12.2	−28.9
	F3	14.8 ± 1.1 a	25.3 ± 0.9 b	25.9 ± 1.3 b	−70.9	−75.0
	F4	58.2 ± 3.0 a	48.5 ± 2.8 b	46.3 ± 3.9 b	16.7	20.4

* Fractions: exchangeable and acid soluble (F1), reducible (F2), oxidizable (F3), and residual (F4). ** A negative efficiency (%) means an increase in a given metal in an individual fraction in washed soil compared to the contaminated soil. Different letters indicate significant differences in metal concentration in a given fraction between the unflushed and flushed soils (ANOVA followed by Tukey’s honest significant difference test, *p* < 0.05).

**Table 4:** Comparison of selected soil characteristics before and after soil flushing with TA (mean ± SD, *n* = 3).

Characteristic	Unit	Unflushed Soil	Flushed Soil **	
			0.5 mL/min	1.0 mL/min
pH	-	8.2 ± 0.4a	6.8 ± 0.2b	6.7 ± 0.3b
Electrical conductivity	mS/cm	2.1 ± 0.2a	0.8 ± 0.1b	0.8 ± 0.1b
Organic matter	%	4.1 ± 0.7a	10.4 ± 0.4b	10.8 ± 0.6b
Cation exchange capacity	cmol/kg	47.8 ± 2.7a	39.0 ± 3.1b	40.0 ± 1.6b
Total Cd	mg/kg	16.0 ± 1.3a	4.6 ± 0.6b	2.3 ± 0.5c
Total Cu		515.8 ± 8.9a	387.9 ± 7.4b	366.7 ± 5.8c
Total Ni		245.1 ± 3.8a	135.1 ± 4.1b	136.8 ± 5.9b
Total Pb		919.1 ± 14.6a	776.7 ± 8.4b	778.5 ± 9.6b
Total Zn		865.8 ± 10.1	665.8 ± 12.4	638.9 ± 15.6
MF_Cu_ *	%	51.3 ± 3.3a	26.7 ± 2.0b	18.7 ± 1.7c
MF_Ni_		68.3 ± 2.6a	59.7 ± 3.1b	54.8 ± 4.3b
MF_Cd_		86.6 ± 4.1a	62.6 ± 3.4b	67.7 ± 4.2b
MF_Pb_		34.1 ± 1.3a	5.3 ± 0.3b	5.5 ± 0.6b
MF_Zn_		67.8 ± 3.5a	52.4 ± 2.8b	49.6 ± 3.1b

* MF, mobility factor. ** SFT = 30 h. Different alphabetical letters indicate significant differences for a given characteristic between the unflushed and flushed soils (ANOVA followed by Tukey’s honest significant difference test, *p* < 0.05).

## Data Availability

The data presented in this study are available on request from the corresponding author.
